# Practical Departments

**Published:** 1905-12-09

**Authors:** 


### PRACTICAL DEPARTMENTS.

### NEW PATENT HOSPITAL WARD LAMP.

(The Birmingham Guild of Handicraft, Limited, 45 Great Charles Street, Birmingham.)

We have received one of these new ward lamps from the Birmingham Guild of Handicraft. The instrument is a very much improved form of the old familiar bell light. It is made in nickel plate with aluminium shade and ebony handle, and is provided with a bracket which may be affixed to the wall or may be used as a stand for the lamp, according to the requirements of the moment. Apart from its neat and serviceable appearance, the lamp has two material advantages over any other lamp designed for the same purpose that we are acquainted with. These are: firstly, the bracket, which has been mentioned, and on which the lamp may be placed when a movable light is not needed or when there is no one by to manipulate it; and, secondly, the detachable handle which may be slipped off and boiled if and when necessary. The great advantage of this, not only in a hospital for infectious diseases but in an ordinary hospital, is obvious. The instrument is superior to any that we have seen designed for the same purpose, and we are glad to recommend it as a desirable, if not, indeed, an essential part of the equipment of a modern hospital ward. The price is 21s.

### THE MARCONI CO.'S X-RAY AND HIGH FREQUENCY APPARATUS.

The Marconi Telegraph Co., of 18 Finch Lane, London, E.C., who have been obliged to go very deeply into the question of the large-spark induction coil for use with wireless telegraphic apparatus, have utilised their experience in this branch of the subject in the manufacture of coils and other apparatus for electro-medical purposes. The coil that is used for wireless telegraphy is suitable for *x*-rays and for high-frequency apparatus, the requirements in the matter of construction being the same in the two cases. The Marconi Co. have also added other apparatus. With their induction coils they supply a condenser regulator, which should be of great use. They make all kinds of interrupters for their coils, and they supply the different forms of *x*-ray tubes, with shields, stands, etc., and also fluorescent screens of various patterns. They make a portable *x*-ray set, with coil, batteries, and all accessories, mounted on a wheeled trolley, for hospital use. The Company make the usual high-frequency apparatus, as well as a special portable set, in which the copper resonator is suspended under the table on which the remainder of the apparatus is mounted. They also make switchboards for taking current from the town electric service for medical work and for other purposes.

